# Gastric Parastomal Hernia: A Case Report of a Rare yet Fascinating Clinical Entity

**DOI:** 10.7759/cureus.4886

**Published:** 2019-06-11

**Authors:** Abdul Waheed, Paul E Zeller, Patrick J Bishop, Sara K Robinson, Faiz Tuma

**Affiliations:** 1 General Surgery, Brandon Regional Hospital, Brandon, USA; 2 General Surgery, Central Michigan University College of Medicine, Saginaw, USA

**Keywords:** gastric parastomal hernia, parastomal hernia, end colostomy, end ileostomy

## Abstract

A 58-year-old female with the prior history of diabetes mellitus (DM) presented with nausea, malaise, and abdominal pain of two days duration. Also, in the past, she was treated for a necrotized rectum from a retroperitoneal infection leading to a colostomy in the left lower quadrant (LLQ) of the abdomen. The physical examination findings were highly suggestive for a parastomal hernia. As a part of her workup and treatment, the initial abdominal CT demonstrated the presence of the gastric contents into the hernia sac leading to the gastric obstruction. The patient responded well to the conservative management using nasogastric (NG) suction, intravenous (IV) line maintenance, clinical assessment, frequent vital sign monitoring, and initiating the nothing per oral (NPO) regimen. Following the successful conservative approach, the patient opted to undergo surgical treatment in the future. This case report and associated literature search represent a rare case of a parastomal hernia with protruding gastric contents, which was successfully treated with conservative management.

## Introduction

A parastomal hernia is a common late complication of ostomy creation, permitting gastrointestinal contents to protrude through an ostomy site [1, 2]. The overall incidence of parastomal hernia in the United States (US) is reported as ranging from 0 to 50%, with end-colostomy (EC) having the highest incidence rate of 4.0-48.1% [3]. Generally, the parastomal hernia sac contains omentum, a portion of the small intestine and colon; however, occasionally, the gastric contents can also extend into the sac [4].

Likewise, the clinical presentation of patients with a gastric parastomal hernia (GPH) depends on the functional status of the gastric contents in the sac [5]. Patients with non-necrotic gastric tissue in the GPH may present with mild abdominal discomfort, slight distension, nausea, and constipation, while patients with incarcerated gastric contents present with severe symptoms including but not limited to severe abdominal pain, acute abdomen, fever, and septicemia [5].

Furthermore, CT of the abdomen is the most widely used diagnosed test for the diagnosis of the GPH [6, 7]. Although the management of the GPH is a clinical dilemma, nevertheless, the majority of the physicians prefer initial conservative management followed by the surgical treatment to correct the anatomical defect [3].

Considering the rarity of this condition, we performed a comprehensive literature search (Table 1) and surprisingly, to the best of our knowledge, most of the GPH literature published so far is limited to only a few case reports. The current case report and literature search is a valuable addition to the limited available literature on this rare condition.

## Case presentation

A 58-year-old woman with a long-standing history of the type 2 diabetes mellitus (DM) presented to the emergency department (ED) with a two-day history of nausea, vomiting, and moderate to severe burning abdominal pain. She additionally noted increased liquid consistency of her stools as well as increased output from her colostomy bag. Her past medical history included a severe necrotized rectum from a retroperitoneal infection requiring a colostomy in left lower quadrant (LLQ) abdomen - also, her family and social history were insignificant.

At the initial presentation, she was afebrile with a blood pressure of 147/77 mmHg, a heart rate of 93 beats/minute, a respiratory rate of 19 breaths/minute, and an oxygen saturation (SpO2) of 97%. Physical examination revealed moderate generalized abdominal tenderness without the signs of peritonitis. Also, a well-appearing LLQ ostomy site without any signs of ischemia and a bulge around the stoma was noticed. Laboratory findings were unremarkable except for an elevated glucose level and her initial white blood count (WBC) of 11.4 × 10^9^/liter with 70.5% of granulocytes.

**Figure 1:**
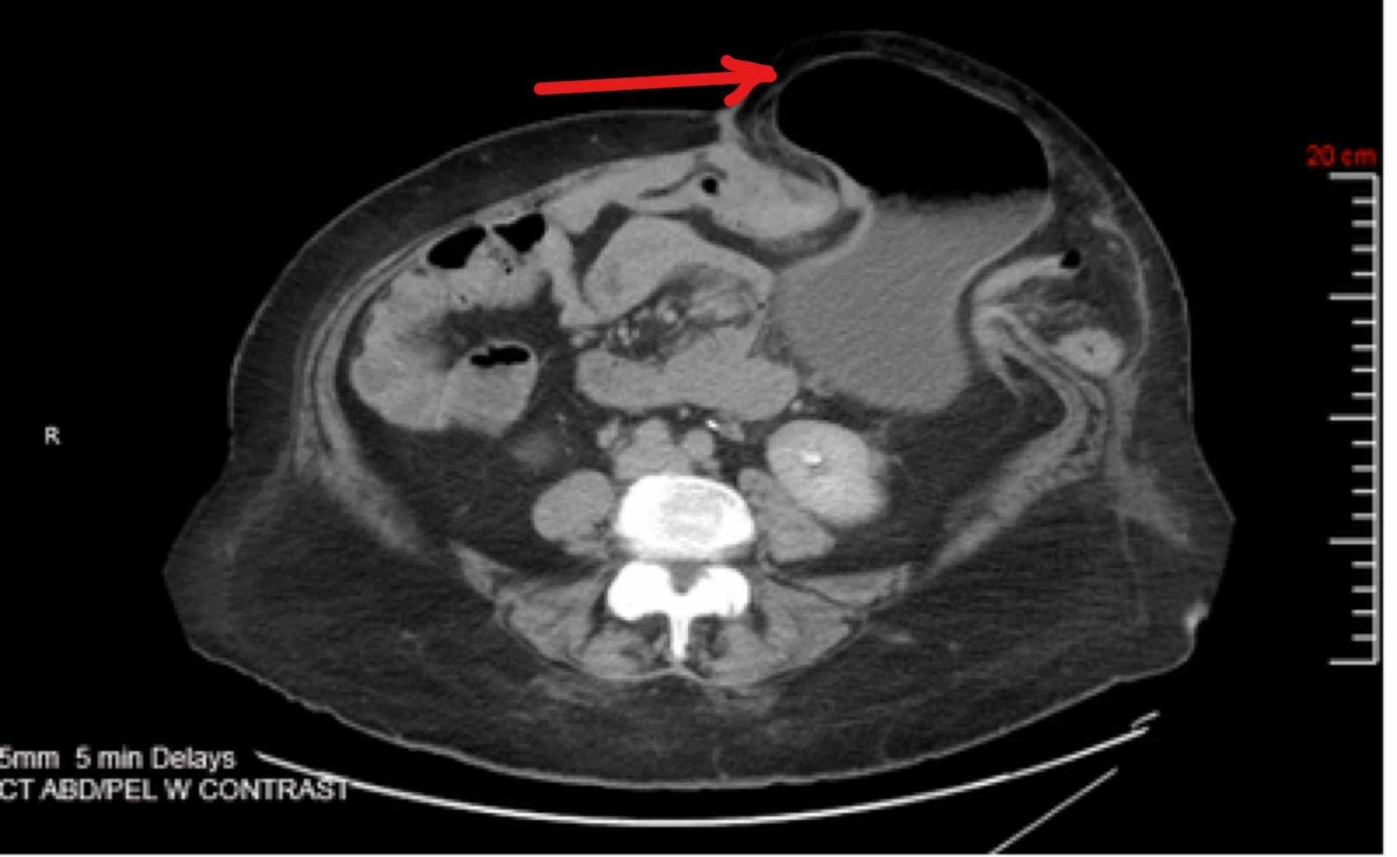
CT abdomen and pelvis CT abdomen demonstrating a large parastomal hernia. There is a portion of the stomach, small bowel, colon, and mesentery within the hernia sac (Arrow indicating the location of the hernia).

**Figure 2:**
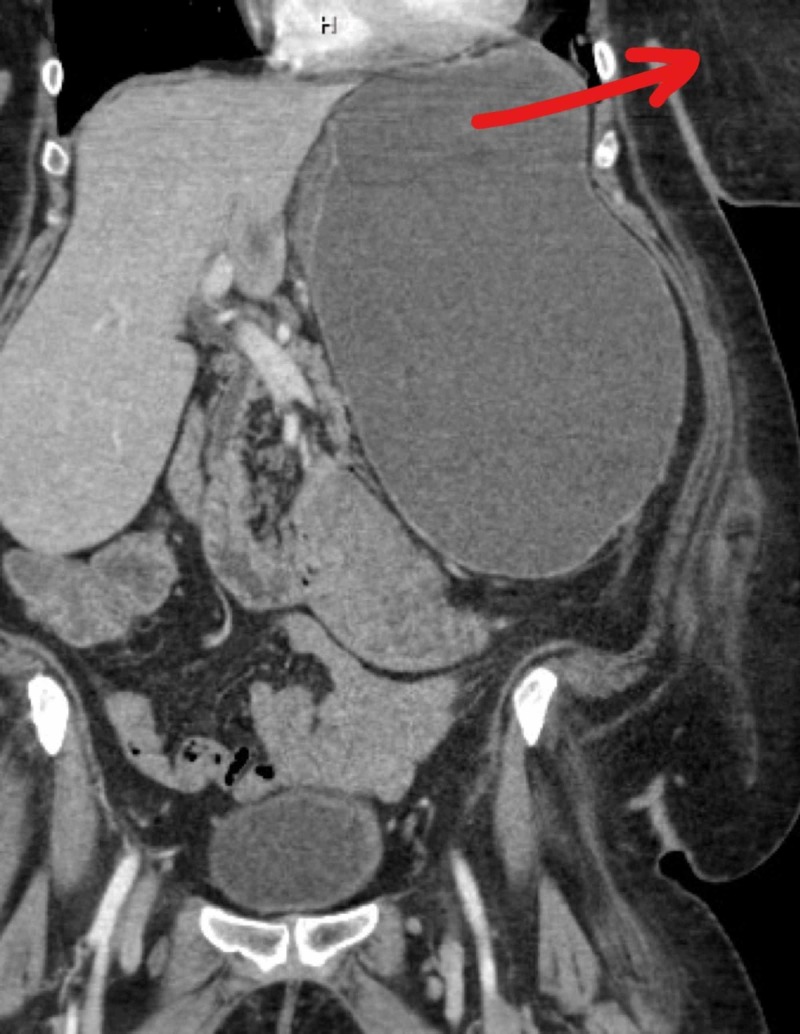
CT abdomen and pelvis CT scan abdomen, coronal view showing large parastomal hernia (Red arrow indicating location of the sac).

Based on the findings of the physical examination, a CT of the abdomen and pelvis was performed, which demonstrated a parastomal hernia causing a partial small bowel and gastric obstruction. Likewise, the parastomal hernia contained a portion of the stomach, small intestine, colon, and mesentery (Figures 1, 2), confirming a diagnosis of the GPH. Moreover, given the clinical presentation and radiological results, a management strategy particular for this patient was developed.

Initially, a conservative approach including an NG tube insertion, IV hydration, serial clinical assessment, and frequent vital signs monitoring, was initiated. Also, based on the patient’s clinical condition, the nothing per oral (NPO) regimen was ordered. The patient promptly started responding to the conservative management on day 1 with the improvement of her symptoms. On day 2, the patient felt much better with the improvement in the abdominal pain and no nausea and vomiting. At this point, the NG tube was removed, and her diet was gradually progressed to a soft food diet.

The additional monitoring of the patient was fairly gratifying. She tolerated the soft diet without any difficulty, other discomforts, nausea, and vomiting. To evaluate the status of her intestinal obstruction, the abdominal X-ray (XR) was ordered, which showed no further evidence of a small bowel obstruction. In the meanwhile, the alternative plan of surgical intervention was also discussed with the patient. The patient showed progressive clinical improvement over the following few days and desired a follow-up appointment with her surgeon after the discharge from the hospital. She was discharged with a plan to follow up with her surgeon from an outside hospital to discuss surgical treatment options for the parastomal hernia.

## Discussion

A parastomal hernia is a common complication following EC (4.0-48.1%), end ileostomy (EI) (1.8-28%), loop colostomy (LC) (0-30.8%), and loop ileostomy (LI) (0-6.2%) [2, 3]. Typically, small intestine, large intestine, and omentum are the most common organs protruding into the hernia sac; however, very rarely stomach can also herniate into the sac [2, 4]. GPH is an extremely infrequent clinical entity usually affecting the elderly female population after the 5th decade of life [7]. The patient presented in the current case report is also a female in the 6th decade of her life.

In an effort to better understand the rarity of the gastric herniation into the hernia sac, Ellingson et al. in a case report reported that stomach is usually held tightly into the abdominal cavity by the numerous ligaments [8]. They likewise added that besides ligaments, the esophagus and diaphragm also hold the stomach superiorly, while the duodenum anchors it inferiorly, which further adds into the decreased mobility of the stomach [8]. Moreover, Asadov et al. in a case report also hypothesized that a facial defect in the abdominal wall must coexist, which can adequately lax the gastric ligaments and allow the gastric contents to protrude into the hernia sac [7]. This mechanism possibly explains the development of GPH in our patient, as she also had a prior colostomy in the LLQ of the abdomen.

Additionally, the clinical presentation of GPH varies according to the extent of gastric involvement [7]. Mostly, all the GPH patients present with the partial or complete gastric outlet obstruction [7]. Patients with the partial obstruction usually present with the symptoms including mild to moderate abdominal pain, nausea, vomiting, and signs of the obstruction, while strangulation, irreducible hernia, perforated stomach, gastric emphysema, and septicemia are most likely in patients with complete and incarcerated hernia [9]. The patient in the current case report presented with moderate abdominal discomfort, nausea, vomiting, and abdominal distension, which suggested the partial obstruction of the stomach.

Although the diagnosis of the GPH is mostly established on the high clinical suspicion and finding on the physical examination, nevertheless, the advancement in radiological imaging has allowed a better delineation and improved detection of this rare entity. The abdominal CT scan has been frequently used not only for the detection of parastomal hernia and its contents but also it adds in detecting the complications associated with the GPH including gastric perforation and emphysema [1, 7]. Although, in our patient, CT scan of the abdomen was the imaging method of choice for the confirmation of the GPH; however, in few case reports other imaging studies including fluoroscopic examination of upper GI tract have also been used for the diagnosis of the GPH [7].

Furthermore, the treatment of the uncomplicated partial GPH is usually conservative, followed by the elective surgical intervention [1, 10]. In cases of life-threatening incarcerated GPH, the emergent surgical intervention is usually performed [11, 12]. The options for the surgical hernia repair in the GPH range from open surgery with or without stomal transposition, with or without prosthesis placement including either onlay method of supra-aponeurotic placement, a sublay method of placing the mesh deep in the wall or between two aponeurotic planes, or the intra-abdominal mesh placement techniques [7, 13]. The patient in our case report successfully responded to the conservative management protocol. The surgical option was delayed because the patient opted for the hospital discharge.

To expand our knowledge regarding the rarity of this clinical entity, a Boolean Logic literature search of the PUBMED database was conducted using a combination of the keywords including “parastomal hernia”, “peristomal hernia”, “incarcerated peristomal hernia”, “colectomy”, “transverse colectomy”, “gastric hernia”, “stomach” and “gastric”. A total of 13 results were found from the PUBMED database, which was further analyzed. Out of these 13 results, only 11 case reports were identified as having parastomal hernia with the gastric contents (Table 1).

**Table 1:** Literature search from the PUBMED database using a combination of various keywords. F = Female; EI = End ileostomy; EC = End colostomy; APR = Abdominoperineal resection; PPC = Panproctocolectomy; LC: Loop colostomy.

Author/publication year	Age (years)	Gender	Previous surgery	Ostomy type	Management
Eastment and Burstow [1] (2018)	91	F	Total colectomy	EI	Conservative gastric decompression
Vierstraete et al. [11] (2018)	69	F	Pelvic exenteration	EC	Laparotomy and mesh herniorrhaphy
Bull et al. [14] (2019)	85	F	LC	EI	Laparotomy, colostomy excision, and herniorrhaphy
Barber-Mille et al. [6] (2014)	69	F	Hartmann’s procedure	EC	Laparotomy with stomal reposition and mesh herniorrhaphy
Marsh and Hoejgaard [5] (2013)	81	F	Rectal resection	EC	Laparotomy with gastric repair and stomal transposition
Ramia-Angel et al. [10] (2012)	64	F	APR	EC	Conservative management with the gastric decompression and gastroscopy
Bota et al. [4] (2012)	41	F	PPC	EI	Laparotomy and mesh herniorrhaphy
Ilyas et al. [12] (2012)	93	F	Hartmann’s procedure	EC	Laparotomy and herniorrhaphy without mesh
McAllister and D'Altorio [15] (1991)	91	F	Hartmann’s procedure	EC	Laparotomy with stomal transposition and herniorrhaphy without mesh
Ellingson et al. [8] (1993)	77	F	Hartmann’s procedure	EC	Laparotomy and herniorrhaphy without mesh
Figiel and Figiel [9] (1967)	76	F	Transverse colostomy	EC	Laparotomy and herniorrhaphy without mesh

## Conclusions

GPH is a rare clinical finding mostly affecting female after the 5th decade of life with a previous history of colostomy or ileostomy formation. A high degree of clinical suspicion along with the use of CT scan abdomen is the key in making a timely diagnosis. The conservative management should be promptly initiated in a patient with uncomplicated GPH, while the urgent surgical treatment should be reserved for the life-threatening cases. Also, large scale institutional studies should be carried out in order to better understand this rare disease.
